# Apoptin mediates mitophagy and endogenous apoptosis by regulating the level of ROS in hepatocellular carcinoma

**DOI:** 10.1186/s12964-022-00940-1

**Published:** 2022-09-01

**Authors:** Yiquan Li, Chao Shang, Zirui Liu, Jicheng Han, Wenjie Li, Pengpeng Xiao, Nan Li, Shanzhi Li, Zhiru Xiu, Gaojie Song, Yaru Li, Ningyi Jin, Jinbo Fang, Xiao Li, Yilong Zhu

**Affiliations:** 1 Academician Workstation of Jilin Province, Changchun University of Chinese Medicine, No. 1035, Boshuo Road, Jingyue Economic and Technological Development Zone, Changchun, 130117 Jilin People’s Republic of China; 2 Changchun Veterinary Research Institute, Chinese Academy of Agricultural Sciences, Changchun, 130122 People’s Republic of China; 3 Institute of Virology, Wenzhou University, Wenzhou, 325035 People’s Republic of China; 4 Jiangsu Co-Innovation Center for Prevention and Control of Important Animal Infectious Diseases and Zoonoses, Yangzhou, 225009 People’s Republic of China

**Keywords:** Apoptin, Human liver cancer, Apoptosis, Autophagy, ROS

## Abstract

**Background:**

Apoptin, as a tumor-specific pro-apoptotic protein, plays an important anti-tumoral role, but its mechanism of autophagy activation and the interaction between autophagy and apoptosis have not been accurately elucidated. Here, we studied the mechanism of apoptin-induced apoptosis and autophagy and the interaction between two processes.

**Methods:**

Using crystal violet staining and the CCK-8 assay, we analyzed the effect of apoptin in the inhibition of liver cancer cells in vitro and analyzed the effect of inhibiting liver cancer in vivo by establishing a nude mouse tumor model. Flow cytometry and fluorescence staining were used to analyze the main types of apoptin-induced apoptosis and autophagy. Subsequently, the relationship between the two events was also analyzed. Flow cytometry was used to analyze the effect of ROS on apoptin-mediated apoptosis and autophagy mediated by apoptin. The effect of ROS on two phenomena was analyzed. Finally, the role of key genes involved in autophagy was analyzed using gene silencing.

**Results:**

The results showed that apoptin can significantly increase the apoptosis and autophagy of liver cancer cells, and that apoptin can cause mitophagy through the increase in the expression of NIX protein. Apoptin can also significantly increase the level of cellular ROS, involved in apoptin-mediated autophagy and apoptosis of liver cancer cells. The change of ROS may be a key factor causing apoptosis and autophagy.

**Conclusion:**

The above results indicate that the increase in ROS levels after apoptin treatment of liver cancer cells leads to the loss of mitochondrial transmembrane potential, resulting in endogenous apoptosis and mitophagy through the recruitment of NIX. Therefore, ROS may be a key factor connecting endogenous apoptosis and autophagy induced by apoptin in liver cancer cells.

**Graphical abstract:**

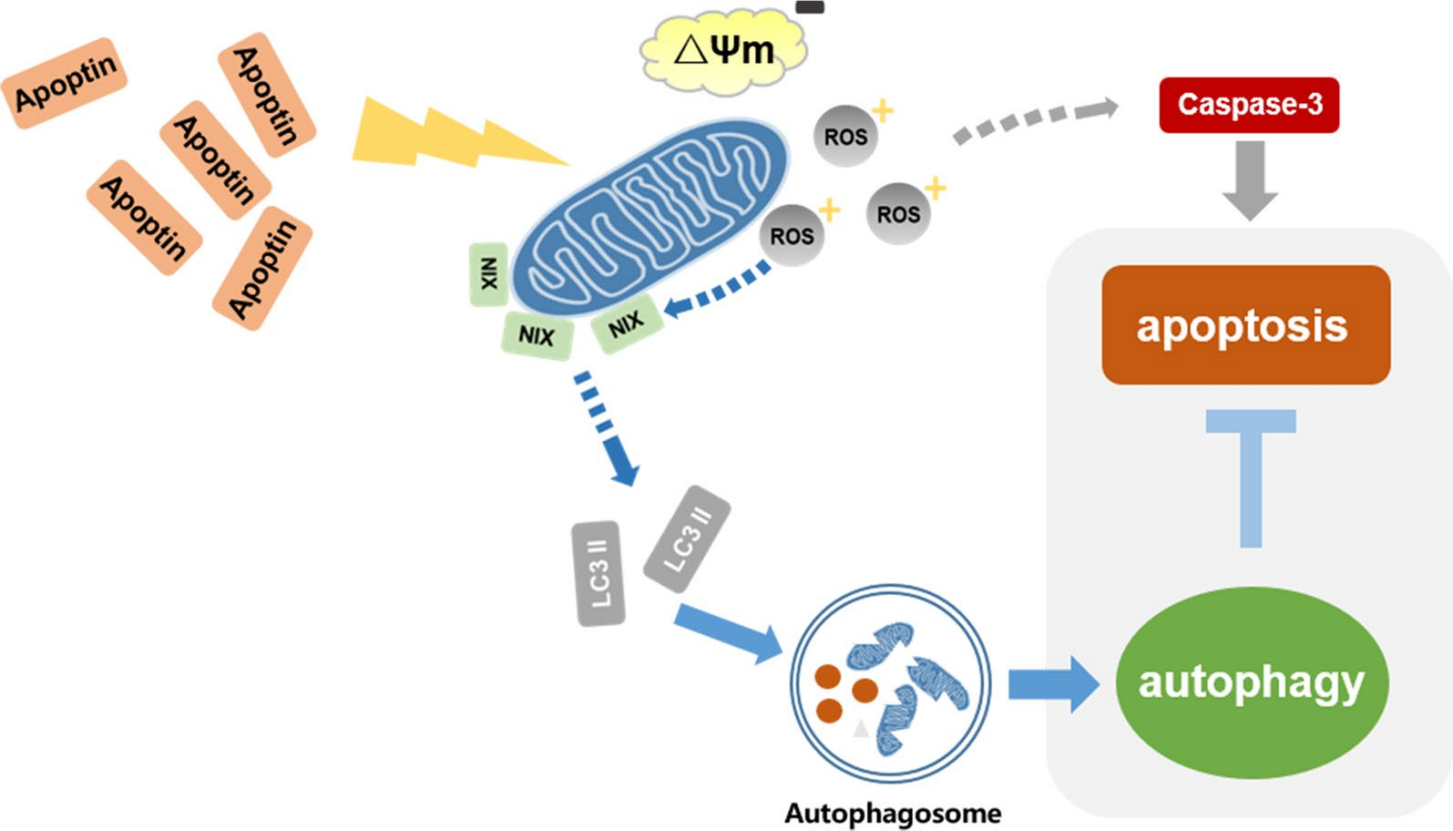

**Video abstract**

**Supplementary Information:**

The online version contains supplementary material available at 10.1186/s12964-022-00940-1.

## Background

Liver cancer is the third largest cause of cancer-related death in the world. In February 2020, the International Agency for Research on Cancer (IARC) of the World Health Organization reported that the number of newly diagnosed cancer cases in the world is 19.3 million. This number continued to increase from the reported 18.1 million cases in 2018 and the number of cancer deaths stands at the million mark and is expected to reach 10 million [1]. From the 19.3 million new cancer cases worldwide, there were 4.57 million new cases of cancer in China, accounting for 23.7% of the global total. As China is the most populous country in the world, the number of new cancers cases far exceeds that of other countries in the world. Currently, surgical resection is still one of the most effective methods to treat this disease. However, the traditional treatments at present, are still unsatisfactory.

With the advances in molecular biology, cell biology, and virology, gene therapy has become a new type of cancer treatment. Oncolytic viruses have shown great advantages as they have the dual functions of viral therapy and gene therapy, and are expected to become an effective method for the treatment of hepatocellular carcinoma (HCC) [2]. The chicken anemia virus (CAV) genome contains three partially overlapping open reading frames, that produce a polycistronic non-spliced RNA encoding three different proteins. Among these, VP3 is the functional protein of CAV and is associated with virus virulence. It is a pathogenic protein that can induce apoptosis of target cells. The VP3 gene is 366 bp in length, encoding the Apoptin protein. The activity of Apoptin depends on its phosphorylation and relocation in the nucleus of tumor cells, which does not exist in normal cells [3]. The selective apoptotic effect of Apoptin is neither dependent on p53 protein-mediated nor inhibited by the overexpression of Bcl-2, and therefore, it is regarded as a new type of biologically active antitumoral protein, which is expected to improve tumor gene therapy [4–7].

Cell death is a complex process that is carefully regulated. Apoptosis is the first recognized type of programmed cell death (PCD), and its role and regulatory network have gradually become clear. However, apoptosis is not the only process that determines the fate of cell death. Autophagy has been proved to regulate cell death along with apoptosis. In some cases, autophagy inhibits apoptosis, but can also induce cell death, or act together with apoptosis, by serving as a standby mechanism to induce cell death in case of apoptotic defects.

Macroautophagy (the autophagy in this topic specifically refers to macroautophagy) is a high-capacity process, originated from the sequestration of the target protein by a cytosolic double-membrane, the formation of autophagic corpuscles, the formation of autophagic lysosomes, and finally, the degradation of unnecessary internal substances and other components. This process is composed of more than 30 types of proteins and regulatory factors [8, 9]. The substrate P62 (Sequestosome-1) is a specific substrate of autophagy that can mediate selective autophagy, such as mitophagy. The autophagic flux was measured by examining differences in the levels of p62, LC3-II, and other autophagy substrate proteins in the presence and absence of lysosomal inhibitors [10].

Selective autophagy can specifically recognize and degrade substrates, which can be protein complexes, damaged organelles, and invading microorganisms. Mitophagy is a form of intracellular autophagy that plays an important pro-survival role in a variety of pathophysiological conditions. Changes in mitochondrial permeability transition (MPT) play important roles in mitophagy, and mitochondrial depolarization is directly related to MPT. During mitophagy, depolarized mitochondria are first encapsulated into autophagosomes, and then fused with lysosomes to form autolysosomes, which degrade mitochondria [11]. Studies have shown the connection between mitophagy and Parkinson's disease. The mutation of the E3 ubiquitin ligase, Parkin, and the mitochondrial kinase Pink1 lead to autosomal recessive inheritance of Parkinson's disease. Under the action of the Pink1 protein, the cytoplasmic Parkin protein translocates to the depolarized mitochondrial surface, which promotes the ubiquitination of mitochondrial proteins, and therefore, induces mitophagy [12, 13]. In addition to Parkin, studies have shown that Nix, BNIP3, and FUNDC1 also play important roles in the process of selective mitophagy [14].

**Fig. 1:**
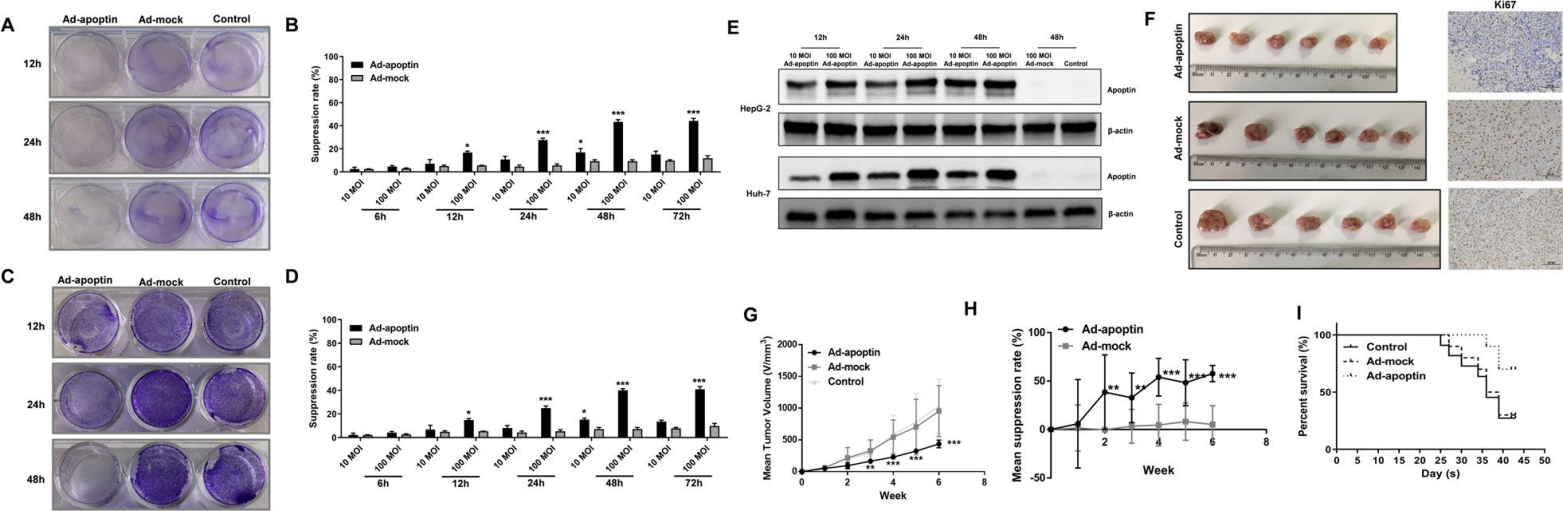
Cytotoxic effect of apoptin on hepatocarcinoma cells. Crystal violet staining and CCK-8 assay analyzes of the inhibitory effect of Ad-apoptin on HepG-2 (**A**, **B**) and Huh-7 (**C**, **D**) cells. (**E**) Expression levels of apoptin in hepatocarcinoma cells infected with recombinant adenovirus at different times. (**G**, **H**) Length and width of xenograft tumors were measured weekly for 6 weeks using Vernier calipers. (**I**) The survival rate of tumor bearing mice was recorded every day for 5 weeks. The data are representative of three independent experiments (n = 3). The unpaired Student’s *t*-test was used. *p < 0.05, **p < 0.01

In our previous study, a recombinant type 5 adenoviral vector overexpressing Apoptin (Ad-apoptin) was constructed (Fig. 1E). This delivery system has been proved to express the protein apoptin stably and effectively in cells [15]. In fact, ectopic expressions using transfection reagents have some disadvantages, including (i) lack of experimental consistency and (ii) decrease of expression due to cytotoxic effects and/or inefficiency and instability of the reagent. In previous studies, we conducted a preliminary study on apoptin-induced apoptosis and autophagy and found that the synergistic action of apoptin and apoptosis causes autophagy in tumor cells.

This study intends to use a human type 5 adenovirus vector expressing the apoptin protein recombinant virus, Ad-apoptin to infect liver cancer cells and investigate whether apoptin has apoptotic and autophagic effects on these cells. The study also explores the relationship between apoptosis and autophagy following induction with apoptin and provides a new theoretical basis for further research in the development of apoptin-based tumor drugs.

## Materials and methods

### Human liver cancer cell lines, recombinant adenovirus, and animals

The liver cancer cell lines, HepG-2 and Huh-7 were cryopreserved by the Key laboratory of Jilin Province for Zoonosis Prevention and Control. The two recombinant adenoviruses (Ad-mock and Ad-Apoptin) were constructed and preserved in our laboratory [15].

Female BALB/c nude mice (4–5 weeks old) were purchased from Beijing vital River Laboratory Animal Technology Co., Ltd., and the animal experiment protocols were approved by the Institutional Animal Care and Use Committee (IACUC) of the Changchun University of Chinese Medicine (Approval No. 2020119). All surgeries were performed under anesthesia. After experiments, the remaining mice were euthanized by intraperitoneal injection of three times the anesthetic dose of sodium pentobarbital (150 mg/kg) for 2–3 min. The euthanasia method followed the AVMA Guidelines for the Euthanasia of Animals.

### Crystal violet staining

The cells were seeded in a 12-well plate and cultured for 24 h. Then the recombinant adenovirus infection (100 MOI) was added. After 12, 24, and 48 h, the cells’ supernatant was discarded, and the wells washed three times with PBS. A total of 600 μL 0.4% crystal violet staining solution was added to each well for 10 min at room temperature, then the solution was discarded, the wells washed three times with PBS, the plate placed in a dry environment, which was followed by photos capture for analysis.

### CCK-8 assay

Cell suspensions were prepared and added to a 96-well plate for culture. The 96-well cell culture plate was taken at 5 different time points (6, 12, 24, 48, and 72 h), and in the supernatant of each well was discarded and the CCK-8 reagent was added, then the plate was incubated at 37 °C with 5% CO_2_ for 2 h in a dark place. After shaking for 10 s, the OD value was measured at 450 nm using a microplate reader.

### Tumor growth in vivo (Xenograft)

HepG-2 cells (5 × 10^6^) were subcutaneously inoculated into the chest of 5-weeks-old BALB/c nude mice to establish a tumor model. The xenografted mice were randomly divided into 3 groups: a 1 × 10^8^ PFU/100 μL Ad-apoptin treatment group, a 1 × 10^8^ PFU/100 μL Ad-mock treatment group, and a control group. The tumors’ size was measured, and survival rate was observed every week. After 42 days of treatment, the animals were euthanized, and each tumor was fixed with formalin. A growth curve was drawn, and the tumors’ volume was calculated:

$${\text{Tumor}}\,{\text{volume}}\,\left( {{\text{mm}}^{3} } \right)\, = \,\left( {{\text{long}}\,{\text{diameter}}\,{\text{of}}\,{\text{tumor}}\, \times \,{\text{short}}\,{\text{diameter}}\,{\text{of}}\,{\text{tumor}}^{2} } \right)/2.$$

$$\begin{aligned} & {\text{Inhibition}}\,{\text{rate}}\,{\text{was}}\,{\text{calculated}}\,{\text{using}}\,{\text{the}}\,{\text{formula}}: \\ & {\text{Tumor}}\,{\text{inhibition}}\,{\text{rate}}\, = \,\left( {1 - {\text{treatment}}\,{\text{group}}\,{\text{tumor}}\,{\text{volume}}/{\text{control}}\,{\text{tumor}}\,{\text{volume}}} \right) \times 100\% \\ \end{aligned}$$

### Immunochemistry (IHC)

Tissue sections were deparaffinized, rehydrated, and incubated in 3% H_2_O_2_ and methanol for 15 min to eliminate endogenous peroxidase activity. The slides were put in 0.01 M sodium citrate buffer (pH 6.0) and incubate at 95 °C for 20 min to perform antigen retrieval. Then the slides were incubated with the primary antibody overnight at 4 °C. After incubating with the secondary antibody for 1 h at room temperature, DAB was used for immunostaining, and the sections were counter-stained with hematoxylin. This step was followed by the analysis of the IHC mean density.

### Hoechst staining assay

The cells were seed in a 12-well plate and cultured for 24 h. Then the recombinant adenovirus infection (100 MOI) was added. After 12, 24, and 48 h, the cells were collected, and then 100 μL Hoechst dye solution was added. The cells were stained for 15 min in the dark, then 10 μl of the stained cell mixture was taken and analyzed by a fluorescence microscope.

### Annexin V-FITC/PI staining assay

The cells were seeded in a 12-well plate and cultured for 24 h. After incubation, the recombinant adenovirus infection (100 MOI) was added. Then the cells were treated with FITC-coupled Annexin-V apoptotic kit for 12 h, 24 h, and 48 h, and apoptosis was detected by FACS flow cytometry.

### Detection of mitochondrial membrane potential

The cells were seeded in a 12-well plate and cultured for 24 h. After culture, the recombinant adenovirus infection (100 MOI) was added. After 12, 24 and 48 h, the cells were collected, and 100 μL JC-1 dye solution was added. The cells were stained for 15 min in the dark, then 10 μL was collected from the stained cell mixture and analyzed using a fluorescence microscope.

The cells were prepared in 96-well plates and incubated at 5% CO_2_ and 37 °C for 24 h. Then the diluted JC-1 solution was added to each well and incubated at 37 °C in the dark for 15 min. The results were determined using microplate reader.

### Western blotting

A whole cell protein extract was prepared with RIPA cell lysate, containing protease inhibitors. The same amount of protein samples was separated on a 10% SDS polyacrylamide gel and transferred to a PVDF membrane. The membrane was blocked with 5% skim milk for 1–2 h, then incubated with the primary antibody overnight at 4 °C. This step was followed by the incubation with the secondary antibody at room temperature for 2 h, and finally, an enzyme-linked chemiluminescence (ECL) detection kit was used. The results were quantitatively analyzed with chemiluminescence and fluorescence imaging systems. The detailed steps were performed as previously described [16].

### Immunofluorescence assay

The cells were seeded in a 12-well plate (with sterile cell slides) and cultured for 24 h. After culture, Ad-apoptin (100 MOI) and different reagents were added, according to different groups. After 48 h, the cells in the wells were fixed with 4% paraformaldehyde for 15 min, treated with 0.5% Triton X-100, blocked with 1% bovine serum albumin (BSA) for 2 h, incubated with primary antibodies overnight at 4 °C, and then washed 3 times with PBS. The Secondary antibodies labeled with FITC or CY3 were incubated for 2 h and the cells were analyzed using a Zeiss confocal microscope.

### LC3 detection assay

The cells were transfected with pEGFP-LC3 for 24 h and then infected with recombinant adenovirus (100 MOI). After 12, 24, and 48 h, the cells were fixed and observed with a fluorescence microscope.

### Lysosomal staining assay

The cells were seeded in a 12-well plate and cultured for 24 h. After culture, the recombinant adenovirus (100 MOI) was added. After 12, 24 and 48 h, the cells were collected, and 100μL LTR (Lyso-Tracker Red) dye solution was added. The cells were stained for 15 min in the dark, then 10 μl of the stained cell mixture was collected and analyzed using a fluorescence microscope.

### TMRM staining assay

The cells seeded in a 12-well plate and cultured for 24 h. After culture, the recombinant adenovirus (100 MOI) was added. After 12, 24, and 48 h, the cells were collected, and 100μL TMRM and Hoechst dye solution were added. The cells were stained for 15 min in the dark, and 10 μl of the stained cell mixture was collected and analyzed using a fluorescence microscope.

### Observation of the co-localization of lysosomes and mitochondria

The cells were seeded in a 12-well plate and cultured for 24 h. After culture, the recombinant adenovirus (100 MOI) was added. After 12, 24, and 48 h, the cells were collected, and 100μL MTG (Mito-Tracker Green) and LTR dye solution were added. The cells were stained for 15 min in the dark, and 10 μl of the stained cell mixture was collected and analyzed using a fluorescence microscope.

### ROS detection

The cells were seeded in a 12-well plate and cultured for 24 h. After culture, the recombinant adenovirus (100 MOI) was added. After 12, 24, and 48 h, the cells were collected, and 100 μL DHR dye solution was added. The cells were stained for 30 min in the dark and flow cytometry was performed to observe the changes in ROS levels.

### Statistical analysis

The statistical analyses were conducted using data from at least three independent experiments, and graph Pad Prism 8 was used for data processing. The data between the two groups were analyzed by random *t*-test. There was significant difference when p < 0.05.

## Results

### Inhibitory growth effect of apoptin in liver cancer cells

Through crystal violet staining and CCK-8 detection, we analyzed the inhibitory effect of apoptin on liver cancer cells (Fig. 1A–D). We found that apoptin can produce significant cytotoxic effects (p < 0.05) in hepatoma cells in a dose- and time-dependent manners. From the CCK-8 experiment, we found that the inhibition rate reaches its peak at 48 h, and that the inhibition rate at 72 h was similar to that at 48 h (45.02% and 42.85%, respectively), with no significant difference. Then we also found that the inhibitory effect of Ad-apoptin at 100 MOI was significantly higher than that at 10 MOI. Obviously, the inhibitory effect is dose and time related.

### Apoptin anti-tumor effect in vivo

We established a subcutaneous tumor-bearing model of HepG-2 cells in nude mice. The results showed that the tumor volume of the Ad-mock group had no obvious change compared with that of the control group, but the tumor volume was significantly reduced after injection of Ad-apoptin, which was significantly different from that of the control group (p < 0.05) (Fig. 1G–H). The Ki67 results showed that the proliferation ability of tumors significantly decreases after injection of Ad-apoptin. We also found that the injection of Ad-apoptin can prolong the survival time of mice and increase the survival rate (Fig. 1I). The above results are basically consistent with the results obtained in *vitro*. Taken together, these results indicate that apoptin had a significant inhibitory effect in *vivo* and in *vitro*, and that it can significantly improve the survival rate of mice.

### Apoptin induces apoptosis through the mitochondrial pathway

**Fig. 2:**
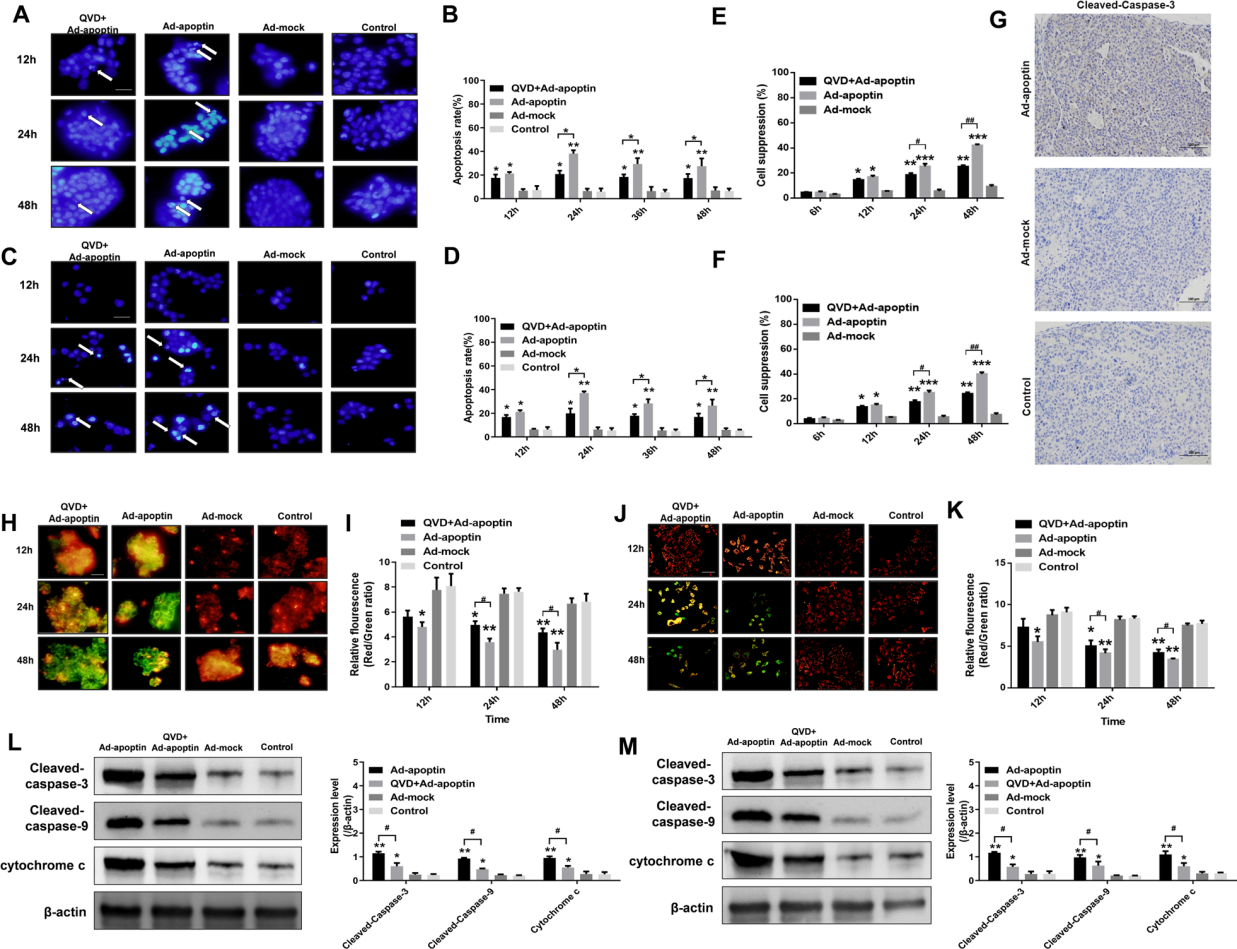
Pathway of apoptin induction of hepatocarcinoma cells’ death. The apoptosis of HepG-2 (**A**, **B**) and Huh-7 (**C**, **D**) cells were detected by Hoechst and Annexin V-FITC/PI staining. After the addition of the caspase inhibitor QVD (20 μM) to the cells infected with Ad-apoptin, the inhibition rate of HepG-2 (**E**) and Huh-7 (**F**) cells were detected by CCK-8 assay. (**G**) Detection of cleaved caspase-3 expression in the xenograft tumor tissues by IHC. JC-1 staining assay analyzes of the changes in mitochondrial membrane potential (MMP) of HepG-2 (**H**, **I**) and Huh-7 (**J**, **K**) cells. (**L**, **M**) Western blot analysis of mitochondrial apoptosis pathway-related protein expression. The scale bar equals 100 µm. Data are representative of three independent experiments (n = 3). The unpaired Student’s *t*-test was used. *p < 0.05, **p < 0.01, ***p < 0.001. (^#^*p* < 0.05, ^##^*p* < 0.01) when compared with Ad-apoptin

To analyze whether apoptin kills liver cancer cells through the apoptotic pathway, we performed Hoechst staining and detected the level of apoptosis (Fig. 2A–D). Hoechst staining showed that cells with apoptin began to die at 12 h, as reflected by the observation of large-scale nuclear fragmentation and over-staining at 24 and 48 h post-infection. No apoptosis was observed in the ad-mock and control groups. From detection results of apoptosis level, the apoptosis level increased from 12 h and reached its peak at 24 h (32.13% for HepG-2 and 26.74% for Huh-7 cells, respectively) (Additional file 1: Fig. S1A-B). After adding the caspase inhibitor QVD, the apoptin-induced apoptosis and the inhibition rate of liver cancer cells significantly decreased (Fig. 2E, F). Immunohistochemical results also showed that apoptin can significantly increase the expression level of caspase-3 (Fig. 2G).

To determine whether apoptin kills liver cancer cells through the mitochondrial apoptotic pathway, we first performed the JC-1 staining test (Fig. 2H–K). After treating the cells with apoptin, the mitochondrial membrane potential gradually decreased. With time, the number of apoptotic cells gradually increased, while the cells were largely depolarized. The mitochondrial membrane potential of the cells that were treated with both Ad-apoptin and QVD was significantly increased, and the ratio of red fluorescence/green fluorescence was significantly higher than that of the Ad-apoptin group. In addition, we also analyzed expression levels of mitochondrial pathway-related proteins, caspase-3, 9, and cytochrome c, and found that expressions of these proteins were significantly increased. Moreover, the addition of QVD inhibited the expression of these proteins (Fig. 2L, M). The above results indicate that apoptin may induce HCC cell apoptosis by activating the mitochondrial apoptotic pathway.

### Changes in autophagy activity

**Fig. 3:**
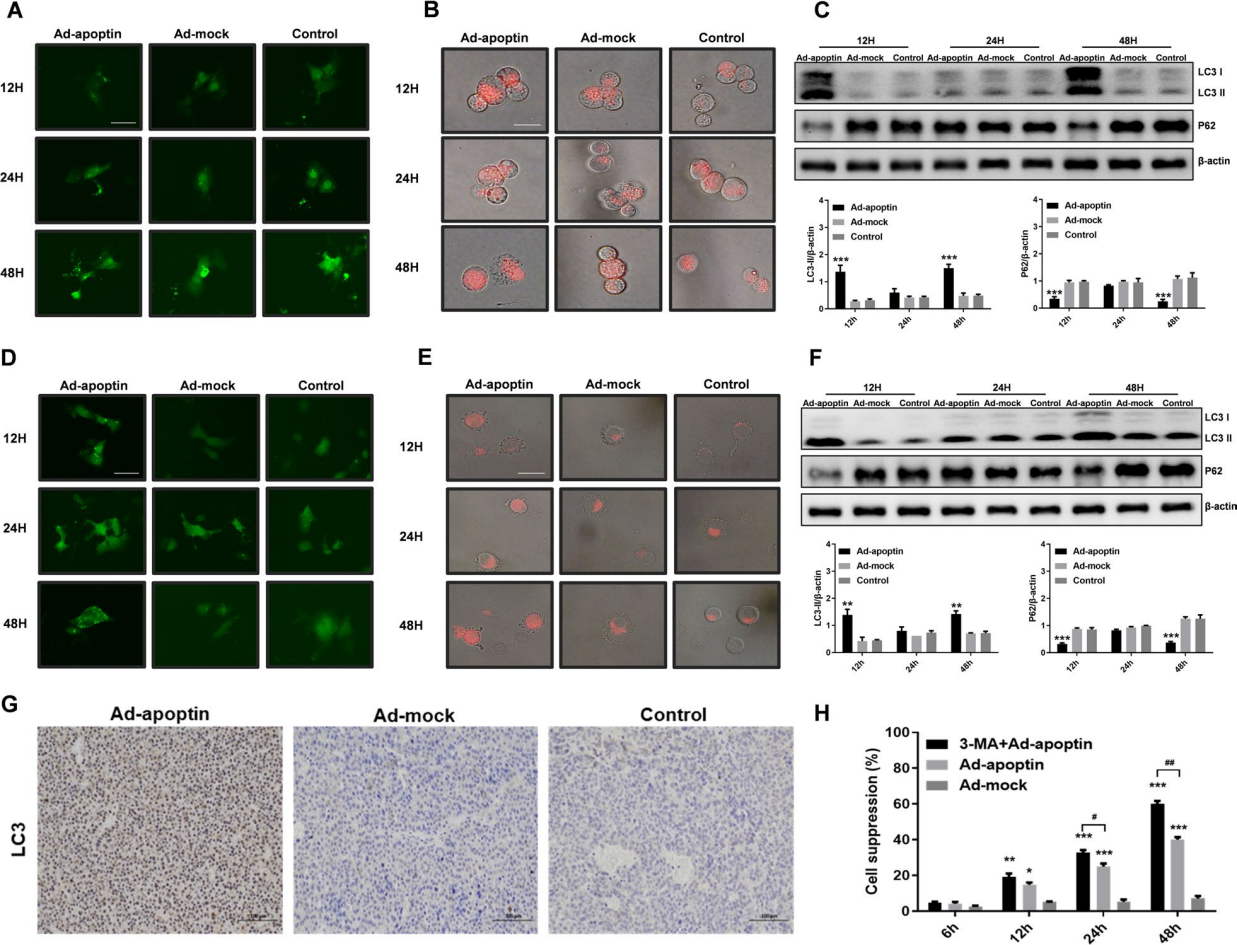
Identification of the effect of autophagy on the apoptin-induced apoptosis of liver cancer cells. Analysis of changes in LC3 fluorescence in HepG-2 (**A**) and Huh-7 (**D**) cells using the LC3-GFP plasmid. After LTR staining in HepG-2 (**B**) and Huh-7 (**E**) cells, the changes in lysosomal fluorescence intensity were observed by fluorescence microscopy. Western blot assay analyzes autophagy and apoptosis-related protein expression levels in HepG-2 (**C**) and Huh-7 (**F**) cells. **G** The expression of cleaved LC3 in the xenograft tumor tissues, was detected by IHC. **H** After the addition of the autophagy inhibitor 3-MA (2 mM) to the cells infected with Ad-apoptin, the inhibition rates of HepG-2 and Huh-7 cells were detected by the CCK-8 assay. The scale bar equals 50 µm. Data were representative of three independent experiments (n = 3). The unpaired Student’s *t*-test was used. *p < 0.05, **p < 0.01, ***p < 0.001. (^#^*p* < 0.05, ^##^*p* < 0.01) when compared with Ad-apoptin

Next, we analyzed whether apoptin affects the level of autophagy after treatment of liver cancer cells. As shown in Fig. 3A–E, the cells in the Ad-apoptin group showed obvious LC3 green fluorescence at 12 h, which remained unchanged at 24 h, and significantly increased at 48 h. Similarly, in lysosomal staining, the results were similar to those of the LC3 experiment. The red fluorescence increased at 12 h, remained unchanged at 24 h, and reached the highest level at 48 h, indicating that apoptin can inhibit the growth of hepatoma cells and affect autophagy.

Subsequently, the levels of autophagy-related proteins were analyzed (Fig. 3C, F, G). After adding Ad-apoptin, the level of LC3 first increased, then decreased, followed by another increase, while the trend of P62 was opposite to that of LC3. The above results indicate that apoptin can affect the level of autophagy in liver cancer cells.

In addition, we analyzed whether the increase in autophagy affects apoptin-mediated killing of the liver cancer cells. The results showed that the inhibition of autophagy can significantly improve the killing effect of apoptin on liver cancer cells, and that the inhibition rate can reach 62.83% at 48 h (Fig. 3H).

### The effect of apoptin-mediated apoptosis on autophagy

**Fig. 4:**
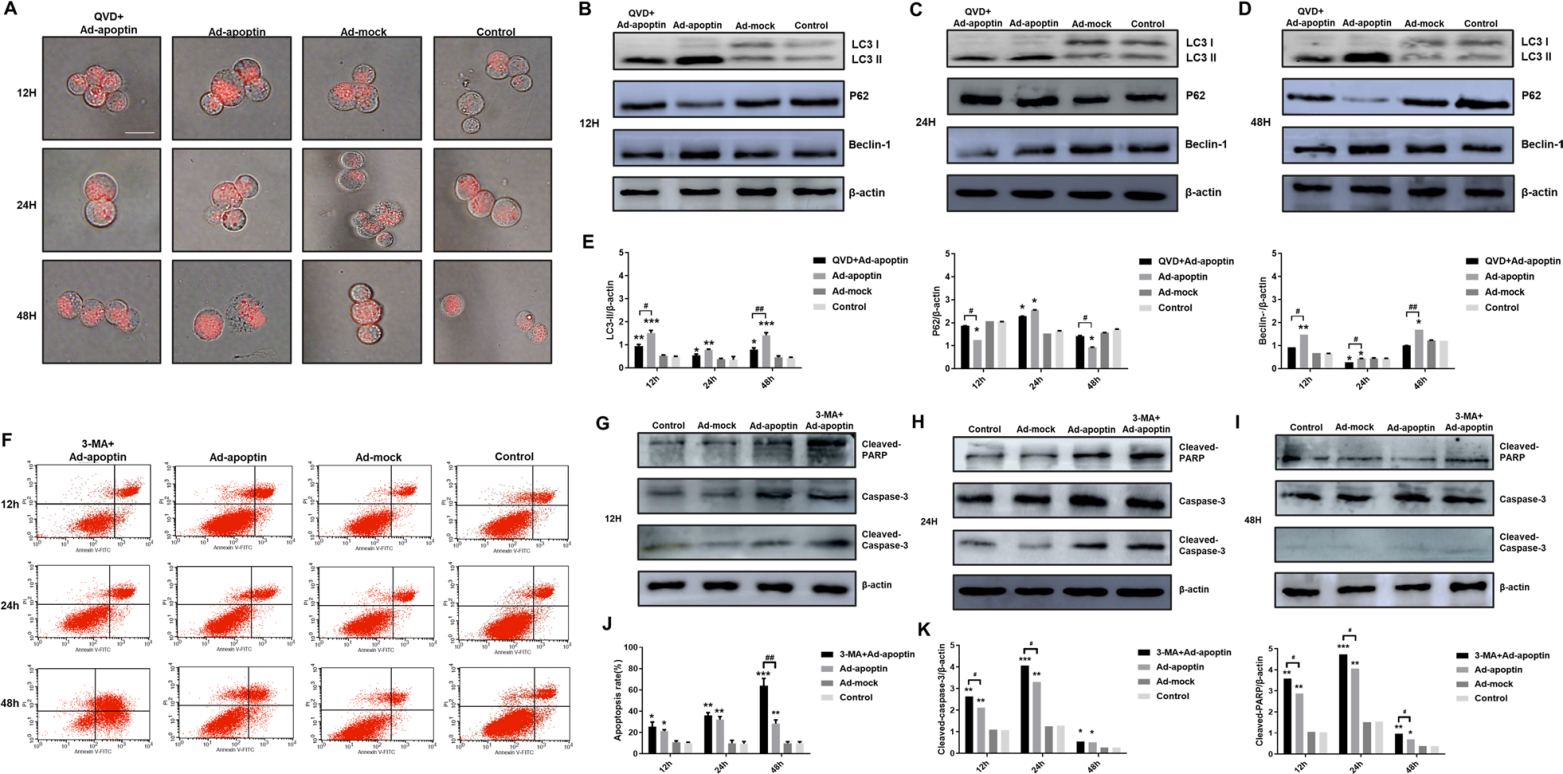
Analysis of the relationship between apoptin-mediated autophagy and apoptosis. **A** After LTR staining in HepG-2 cells, the changes in lysosomal fluorescence intensity were observed by fluorescence microscopy. **B**–**E** Western blot analysis of the expressions of autophagy-related proteins. **F** and **J** Apoptosis analysis using flow cytometry after Annexin-V FITC/PI staining. **G**–**I** and **K** Western blot analysis of the expressions of apoptosis-related proteins. The scale bar equals 50 µm. Data were representative of three independent experiments (n = 3). The unpaired Student’s *t*-test was used. *p < 0.05, **p < 0.01, ***p < 0.001. (^#^*p* < 0.05, ^##^*p* < 0.01) when compared with Ad-apoptin

To analyze whether apoptin-induced apoptosis influences autophagy, liver cancer cells were first stained by LTR (Fig. 4A). After adding QVD, the number of lysosomes was significantly reduced, indicating that the inhibition of apoptosis may significantly reduce the level of autophagy.

Subsequently, autophagy-related proteins were detected (Fig. 4B–E). After inhibiting apoptosis, we found that expression levels of the autophagy-related proteins, LC3-II and Beclin-1, were significantly reduced, while the expression level of p62 was significantly increased (p < 0.05). The above results indicate that decreasing apoptosis can reduce the increase in apoptin-mediated autophagy, and that the higher the level of apoptosis is, the greater the impact will be.

### The effect of autophagy on apoptosis

Next, to analyze whether autophagy influences apoptin-induced apoptosis, we first detected the level of apoptosis (Fig. 4F, J). After inhibiting autophagy, we found that apoptin-induced apoptosis was significantly increased and exceeded the level of apoptosis in the apoptin group alone (p < 0.05). The highest level was 48 h, and the apoptotic rate was 63.54%. The above results indicate that inhibiting autophagy can significantly increase apoptin-mediated apoptosis of liver cancer cells.

Then, the apoptosis-related proteins were detected. After addition of 3-MA, we found that the expression levels of PARP and Caspase-3 significantly increased (Fig. 4G–K). Starting from 12 h, the level of apoptosis-related proteins in the 3-MA + Ad-apoptin group, was constantly higher than that in the Ad-apoptin group (p < 0.05). Once again, the above results show that inhibiting autophagy can significantly increase the level of apoptin-mediated apoptosis.

### Apoptin can cause mitochondrial autophagy

**Fig. 5:**
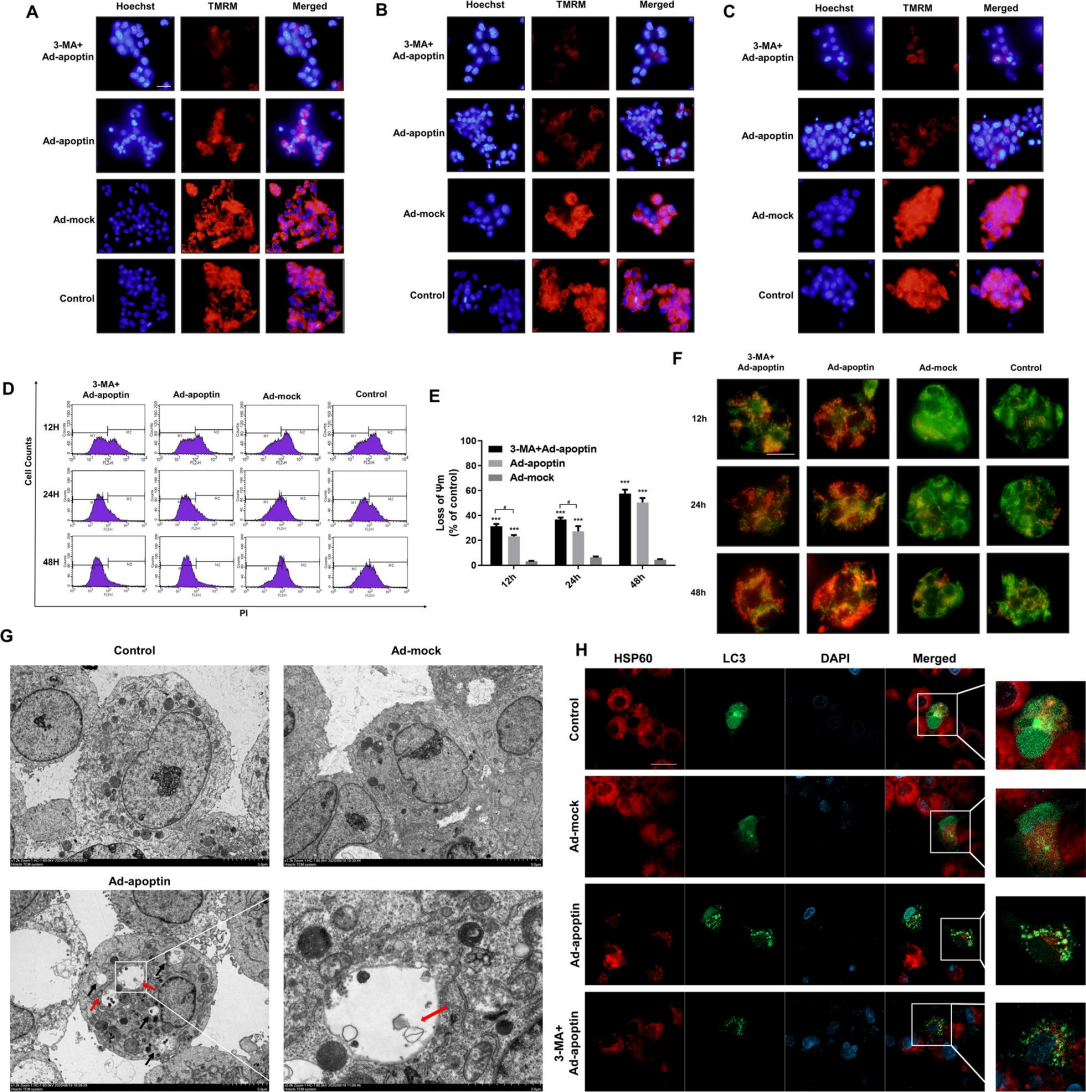
Analysis of the relationship between apoptin-mediated autophagy and mitochondria. **A**–**C** Qualitative analysis of changes in mitochondrial membrane potential by TMRM staining. With the prolongation of time, the mitochondrial membrane potential of the cells in the apoptin group decreased significantly, and the decrease was more obvious after adding 3-MA. **D**, **E** Quantitative analysis of changes in mitochondrial membrane potential by TMRM staining. **F** Analysis of the co-localization of mitochondria and lysosomes by MTG and LTR staining. **G** Formation of autophagosomes and their type of contents observed under an electron microscope. **H** Analysis of the co-localization of LC3 and HSP60 by immunofluorescence staining. The scale bar equals 50 µm. Data were representative of three independent experiments (n = 3). The unpaired Student’s *t*-test was used. *p < 0.05, **p < 0.01, ***p < 0.001. (^#^*p* < 0.05, ^##^*p* < 0.01) when compared with Ad-apoptin

TMRM staining was used to further analyze the effect of autophagy on mitochondria (Fig. 5A–E). At 12 and 24 h, the inhibition of autophagy significantly decreased mitochondrial membrane potential, indicating an aggravation of mitochondrial depolarization (p < 0.05) (Fig. 5D, E). This suggests that the level of mitochondrial depolarization is related to apoptin-induced autophagy.

Next, MTG and LTR staining were used to further analyze the relationship between mitochondria and autophagy (Fig. 5F). After 12 h, the mitochondria green fluorescence decreased, and the lysosomes’ red fluorescence increased. At 24 h, the red fluorescence decreased, and the green fluorescence continued to decrease. After 48 h, we found that the decrease of mitochondrial green fluorescence was the most obvious, and the enhancement of the lysosomes’ red fluorescence was also the most significant. After inhibiting autophagy, both the red and green fluorescence decreased, and the green fluorescence rapidly decreased. Interestingly, the cell areas of the increased red fluorescence and decreased green fluorescence in the Ad-apoptin group, were at the same localization.

Subsequently, electron microscopy showed that there were many autophagosomes in the Ad-apoptin group with damaged mitochondria (Fig. 5G). Then, we analyzed the localization of mitochondrial surface proteins and LC3 and found that there were many co-localizations in the Ad-apoptin group, where the green fluorescence and red fluorescence superimposed generating an orange fluorescence. After adding 3-MA, the co-localization phenomenon was significantly reduced (Fig. 5H). The above results suggest that when apoptin inhibits the proliferation of liver cancer cells, damaged mitochondria are degraded by autophagy.

### ROS regulates apoptin cytotoxic effect on liver cancer cells

**Fig. 6:**
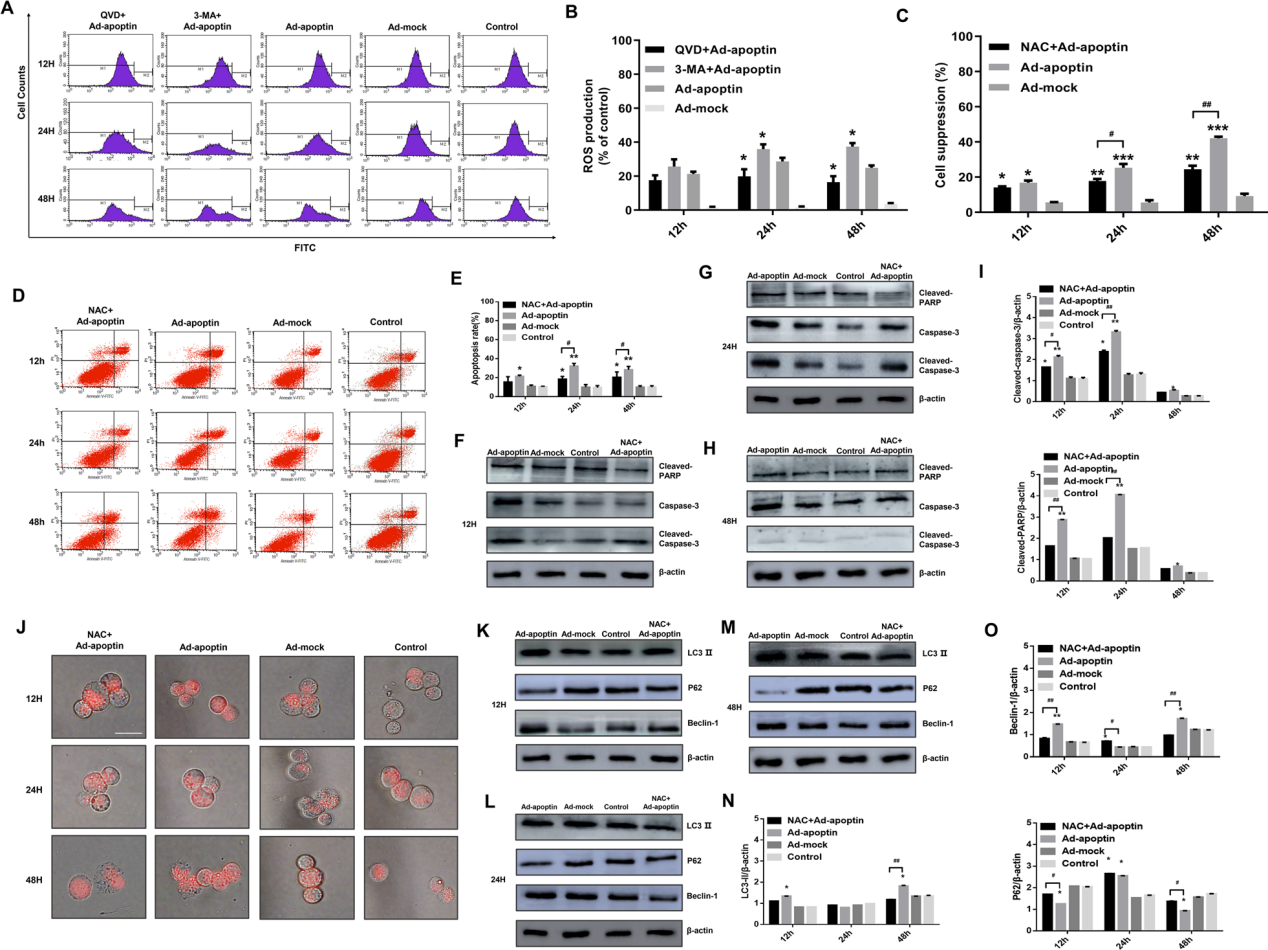
The role of ROS in apoptin-mediated autophagy and apoptosis. **A**, **B** Quantitative analysis of changes in ROS level by DHR staining. **C** After the addition of the ROS inhibitor NAC (10 mM) to the cells infected with Ad-apoptin, the inhibition rate of HepG-2 cells was detected using CCK-8 assay. **D**, **E** Quantitative analysis of changes in apoptosis was conducted by using the Annexin-V FITC/PI assay. **F**, **I**. Western blot analysis of apoptosis-related protein expressions. **J** After LTR staining, the changes in lysosomal fluorescence intensity were observed by fluorescence microscope. **K**–**O** Western blot analysis of the expressions of autophagy-related proteins. The scale bar equals 50 µm. Data were representative of three independent experiments (n = 3). The unpaired Student’s *t*-test was used. *P < 0.05, **p < 0.01, ***p < 0.001. (^#^*p* < 0.05, ^##^*p* < 0.01) when compared with Ad-apoptin

To analyze whether apoptin affects the level of ROS when inhibiting the proliferation of liver cancer cells, we analyzed the level of ROS (Fig. 6A, B). Starting at 12 h, the ROS level in the Ad-apoptin group gradually increased and reached a peak at 24 h. After inhibition of apoptosis, we found that the level of apoptin-induced ROS is reduced to varying degrees. After inhibition of autophagy, we found that the level of ROS was significantly improved (p < 0.05). The above results indicate that apoptin can cause a significant increase in ROS levels, and that this increase is closely related to apoptosis and autophagy.

Subsequently, using the CCK-8 assay, we found that after inhibiting the level of ROS, the inhibitory effect of apoptin on liver cancer cells was significantly reduced (Fig. 6C). This result is similar to the result observed after inhibition of apoptosis. This result indicates that apoptin may induce the death of liver cancer cells by increasing the level of ROS.

### ROS regulates apoptosis and autophagy changes

To analyze whether ROS will affect apoptosis and autophagy, we conducted qualitative and quantitative analysis of autophagy and apoptosis. First, we found that inhibiting ROS can reduce the level of apoptin-mediated apoptosis (Fig. 6D, E). Then the changes in levels of apoptosis-related proteins were analyzed (Fig. 6F–I). By analyzing the level of apoptosis-related proteins, we found that after inhibiting ROS, the expression levels of cleaved-PARP and cleaved-Caspase-3 can be significantly reduced (p < 0.05). The above results indicate that ROS can affect the apoptin-mediated apoptosis of liver cancer cells.

Subsequently, we performed the analysis of lysosomes staining (Fig. 6J) after inhibiting ROS. We found that the apoptin-induced lysosomal red fluorescence gradually decreased and was significantly weaker than that in the Ad-apoptin group at 48 h. Then we analyzed the levels of autophagy-related proteins (Fig. 6K–O). After inhibiting ROS, the expression level of LC3-II and Beclin-1 significantly decreased, while the expression level of p62 significantly increased (p < 0.05). The above results indicate that ROS can also significantly affect apoptin-mediated autophagy of liver cancer cells.

### NIX is a key protein in apoptin-induced mitophagy of liver cancer cells

**Fig. 7:**
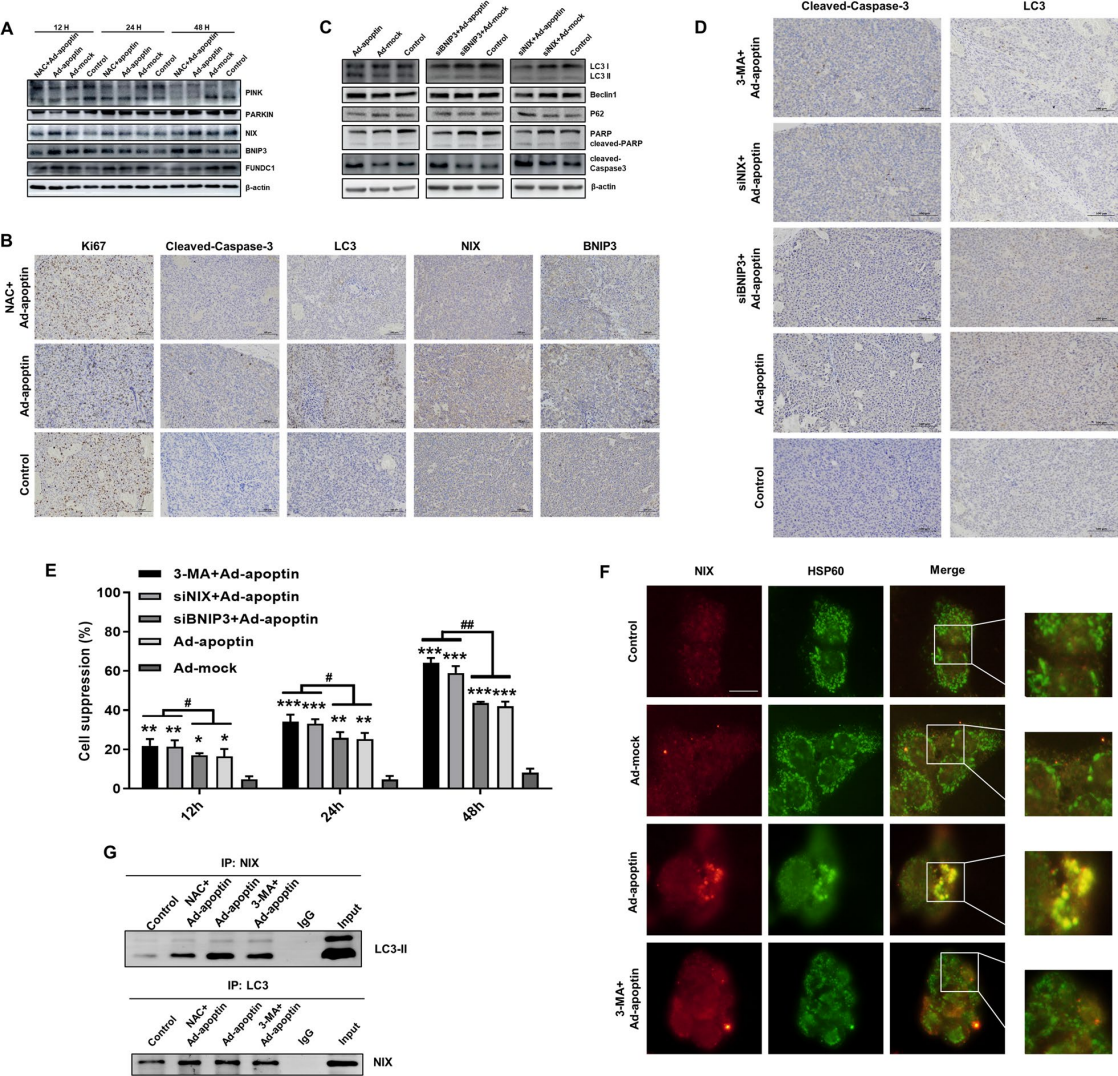
Effect of NIX protein on apoptin-induced mitophagy in liver cancer cells. **A** After addition of the ROS inhibitor, NAC, to the cells infected with Ad-apoptin, the mitophagy related protein level was detected by Western blot assay. **B** Expressions of LC3, cleaved caspase-3, NIX, BNIP3, and Ki67 in the xenograft tumor tissues were detected by IHC. **C** Western blot assay analysis of the changes in the expression levels of apoptosis and autophagy related proteins in MCF-7/ADR cells after silencing NIX and BNIP3. **D** Expressions of LC3 and cleaved caspase-3 in the xenograft tumor tissues were detected by IHC after silencing NIX and BNIP3. **E** After silencing NIX and BNIP3, the inhibition rate of HepG-2 cells was detected by the CCK-8 assay. **F** Analysis of the co-localization of NIX and HSP60 by immunofluorescence staining. **G** Co-immunoprecipitation assay to analyze the interaction between LC3 and NIX. The scale bar equals 20 µm. Data are shown as mean ± SD (*p < 0.05, **p < 0.01, ***p < 0.001) when compared with controls

To further analyze whether mitophagy was induced, we investigated the expression of mitophagy-related proteins. The results showed that the changes in NIX and BNIP3 expressions were similar to those of autophagy-related proteins, and that the decrease of NIX expression was more significant after adding NAC (Fig. 7A). In immunohistochemical experiments, we also found that apoptin can increase the level of apoptosis and autophagy and increase the expressions of NIX and BNIP3. The inhibition of ROS could significantly reduce the levels of apoptosis and autophagy, and the expression level of NIX (Fig. 7B). After silencing NIX and BNIP3, we found that NIX silencing was significantly effective than that of BNIP3 silencing in increasing the levels of cleaved-PARP, cleaved-caspase-3 and p62 and in reducing the levels of LC3 II and beclin1 (Fig. 7C). Similar results were obtained by immunohistochemistry (Fig. 7D). To analyze the roles of NIX and BNIP3 in apoptin-induced autophagy, we analyzed the inhibitory effect of apoptin on liver cancer cells after NIX and BNIP3 were silenced and autophagy inhibited. Similar results were observed whether 3-MA is added, or NIX silenced, but there was no significant difference in the results between the siBNIP3 + apoptin group and the apoptin alone group (Fig. 7E).

We also inhibited autophagy with 3-MA and analyzed the localizations of NIX and mitochondrial membrane proteins (Fig. 7F). The results showed that these proteins colocalize in the Ad-apoptin group. This event was significantly reduced after inhibition of autophagy. Finally, we performed immunoprecipitation experiments, and found that there was an interaction between NIX and LC3 in the Ad-apoptin group, while the interaction between NIX and LC3 was significantly reduced after inhibition of autophagy and ROS (Fig. 7G). The above results indicated that apoptin can mediate the occurrence of mitophagy in liver cancer cells, and that NIX was a key protein in apoptin-induced mitophagy.

## Discussion

Cancer is one of the most serious diseases in the world, and its morbidity and mortality are rapidly rising, which seriously threatens human health. Among all cancers, the morbidity and mortality of liver cancer is also increasing year by year. Hepatocellular carcinoma (HCC) is a highly malignant cancer, characterized by concealment, strong invasion, and rapid progress. Currently, surgery and chemotherapy are still the main treatments for liver cancer. Although chemotherapy is used to treat patients with liver cancer, most patients will relapse in a short time due to chemoresistance. At present, the mechanism of drug resistance is not clear, and treated patients also experience serious toxic reactions during chemotherapy. Although some achievements have been made in precision medicine, such as molecular targeted therapy and immunotherapy, most of these studies are still in the second-line or above clinical stage, due to the studies in small sample size and inconsistencies in the results.

Apoptin is a type of protein that induces a specific apoptosis in tumor cells and is considered as a new anti-tumor biological agent because it is not inhibited by p53 or Bcl-2 overexpression. By studying apoptin, it is also expected to solve the problem of low efficiency, poor specificity, and drug resistance in cancer treatment.

In this study, we first analyzed the cytotoxic effect of apoptin on liver cancer cells through a variety of in *vivo* and in *vitro* experiments. We found that apoptin can significantly cause the death of liver cancer cells, and similar results were previously obtained with other tumor cell types [4, 7, 15, 17–19]. Moreover, we also found that the killing ability of apoptin of liver cancer cells is higher than that observed with other tumor cells, indicating that apoptin may have a higher potential therapeutic effect in the treatment of liver cancer. Interestingly, our *vivo* experiments showed that apoptin can not only inhibit the growth of tumors, but also increase the survival time of mice. Therefore, apoptin can significantly kill liver cancer cells both in *vivo* and in *vitro*.

Studies have shown that apoptin mainly kills a variety of tumor cells through apoptosis [20], such as through the activation of caspases in the human osteosarcoma cell line (Saos-2) to initiate apoptosis [21]. Apoptin also kills Jurkat T cells through a death receptor-independent pathway [22]. In this study, we found that apoptin significantly increases the level of cell apoptosis, and that after adding caspase inhibitors, the killing effect of apoptin on liver cancer cells, was significantly reduced. This result is similar to the results of other studies, showing that apoptin mainly kills various tumor cells through apoptosis [20]. Subsequent analysis of mitochondrial membrane changes revealed that apoptin can significantly damage mitochondria and lead to depolarization of cells. These results show that apoptin can kill liver cancer cells through the endogenous apoptotic pathways.

Autophagy refers to the process of cellular self-digestion marked by the appearance of autophagosomes in the cytoplasm. The proteins and organelles that are damaged, denatured, or senescent are transported to lysosomes, where they are digested and degraded. Studies have shown that tumors are closely related to autophagy, and that many clinical drugs regulating autophagy, play important roles in tumor treatment. Here, we analyzed this event by staining autophagy-related proteins and lysosomes to investigate how apoptin kills HCC cells, while also affecting autophagy which has a protective reaction.

Studies have shown that the stimulation of cells by external factors can cause autophagy, which may lead to apoptosis [23]. The relationship between these two types of programmed cell deaths can be divided into the following categories: (i) Autophagy can alleviate the stress state of the environment on the cell, thereby maintaining the normal function of the cell [24]; (ii) Autophagy can promote apoptosis. By providing the ATP required for death or activation of the caspase activity [25–28]; and (iv) Autophagy can inhibit apoptosis by degrading damaged organelles and maintaining normal cell functions, such as mitochondria function (i.e. mitochondrial autophagy) [29–31].

Here, we studied the relationship between apoptin-induced apoptosis and autophagy. We found that inhibiting apoptosis can reduce the level of apoptin-mediated autophagy in liver cancer cells. In contrast, inhibiting autophagy can increase the level of apoptin-mediated apoptosis of liver cancer cells. In addition, we analyzed the influence of autophagy on mitochondria and found that autophagy may have some influence on the apoptin-induced changes of mitochondrial membrane potential (MMP). Then, we analyzed the relationship between mitochondria and lysosomes. We first discovered that mitochondria and lysosomes co-localized. Subsequently, we observed the co-localization of LC3 and HSP60, and found that these two proteins have strongly co-localizations after treatment with apoptin. Therefore, we preliminarily judged that apoptin may induce autophagy to engulf damaged mitochondria.

As the very important factor in mitochondrial damage, ROS has been identified as an important role playing in this study. We found that apoptin can increase the level of ROS in liver cancer cells, and that ROS can influence changes in apoptosis and autophagy. Similarly, apoptosis and autophagy can also have effects on the level of ROS. This indicates that apoptin can influence apoptosis and autophagy by adjusting the level of ROS.

Next, to further explore whether apoptin induces mitophagy, we investigated the expression of mitophagy-related proteins. The results showed that the changing trend observed for NIX expression was similar to that of the changing trend of autophagy in liver cancer cells induced by apoptin. We also observed that the change of NIX expression may significantly affect the level of autophagy. Subsequently, the inhibition of ROS was also found to significantly decrease the expression level of NIX. Finally, through immunoprecipitation experiments, we found that there was a significant interaction between NIX and LC3, whereas the interaction between NIX and LC3 was significantly reduced after inhibition of autophagy and ROS. All the above results indicate that NIX is a key protein of apoptin-induced mitophagy.

## Conclusions

In summary, apoptin can significantly kill liver cancer cells, mainly through endogenous apoptosis and has a significant impact on autophagy. When apoptin kills liver cancer cells through the induction of apoptosis, autophagy plays a protective role. Through multi-level studies, we found that the apoptin-induced increase in ROS levels leads to the loss of mitochondrial transmembrane potential in liver cancer cells, resulting in apoptosis and mitophagy and the recruitment of NIX protein. The autophagy produced in this process is a protective mechanism that inhibits cell apoptosis. Therefore, our research shows that ROS may be a key factor connecting apoptin-induced apoptosis and autophagy in liver cancer cells.

## Supplementary Information

**Additional file 1**. Flow Graph of Changes in Apoptosis Level of Hepatoma Cells Treated with Apoptin.

## Data Availability

The datasets used and analyzed during the current study are available from the corresponding author on reasonable request.

## Abbreviations

ROS: Reactive oxygen species
PCD: Programmed cell death
CAV: Chicken anemia virus
DRs: Death receptors
FADD: Floationg point addition
Atg: Autophagy-related genes
MOI: Multiple of infection
QVD: Q-VD-OPh
DMEM: Dulbecco’s Modified Eagle Media
NAC: *N*-Acetyl cysteine
LTR: Lyso-Tracker Red
MTG: Mito-Tracker Green

## References

[1] Sung H, Ferlay J, Siegel RL, Laversanne M, Soerjomataram I, Jemal A, Bray F (2021). Global cancer statistics 2020: GLOBOCAN estimates of incidence and mortality worldwide for 36 cancers in 185 countries. CA Cancer J Clin.

[2] Gu D, Kelly TN, Wu X, Chen J, Samet JM, Huang JF, Zhu M, Chen JC, Chen CS, Duan X (2009). Mortality attributable to smoking in China. N Engl J Med.

[3] Backendorf C, Noteborn MHM, Grimm S (2014). Apoptin towards safe and efficient anticancer therapies. Anticancer Genes.

[4] Liu L, Wu W, Zhu G, Liu L, Guan G, Li X, Jin N, Chi B (2012). Therapeutic efficacy of an hTERT promoter-driven oncolytic adenovirus that expresses apoptin in gastric carcinoma. Int J Mol Med.

[5] Zhang M, Wang J, Li C, Hu N, Wang K, Ji H, He D, Quan C, Li X, Jin N, Li Y (2013). Potent growth-inhibitory effect of a dual cancer-specific oncolytic adenovirus expressing apoptin on prostate carcinoma. Int J Oncol.

[6] Qi Y, Guo H, Hu N, He D, Zhang S, Chu Y, Huang Y, Li X, Sun L, Jin N (2014). Preclinical pharmacology and toxicology study of Ad-hTERT-E1a-Apoptin, a novel dual cancer-specific oncolytic adenovirus. Toxicol Appl Pharmacol.

[7] Yang G, Meng X, Sun L, Hu N, Jiang S, Sheng Y, Chen Z, Zhou Y, Chen D, Li X, Jin N (2015). Antitumor effects of a dual cancer-specific oncolytic adenovirus on colorectal cancer in vitro and in vivo. Exp Ther Med.

[8] Levy JMM, Towers CG, Thorburn A (2017). Targeting autophagy in cancer. Nat Rev Cancer.

[9] Mizushima N, Yoshimori T, Ohsumi Y (2011). The role of Atg proteins in autophagosome formation. Annu Rev Cell Dev Biol.

[10] Klionsky DJ, Abdelmohsen K, Abe A, Abedin MJ, Abeliovich H, Acevedo Arozena A, Adachi H, Adams CM, Adams PD, Adeli K (2016). Guidelines for the use and interpretation of assays for monitoring autophagy (3rd edition). Autophagy.

[11] Youle RJ, Narendra DP (2011). Mechanisms of mitophagy. Nat Rev Mol Cell Biol.

[12] Youle RJ, Narendra DP (2010). Mechanisms of mitophagy. Nat Rev Mol Cell Biol.

[13] Narendra D, Tanaka A, Suen DF, Youle RJ (2008). Parkin is recruited selectively to impaired mitochondria and promotes their autophagy. J Cell Biol.

[14] Tolkovsky AM (2009). Mitophagy. Biochim Biophys Acta.

[15] Li X, Liu Y, Wen Z, Li C, Lu H, Tian M, Jin K, Sun L, Gao P, Yang E (2010). Potent anti-tumor effects of a dual specific oncolytic adenovirus expressing apoptin in vitro and in vivo. Mol Cancer.

[16] Li X, Jin N, Mi Z, Lian H, Sun L, Li X, Zheng H (2006). Antitumor effects of a recombinant fowlpox virus expressing Apoptin in vivo and in vitro. Int J Cancer.

[17] Jin J, Zhu Y, Sun F, Chen Z, Chen S, Li Y, Li W, Li M, Cui C, Cui Y (2018). Synergistic antitumor effect of the combination of a dual cancer-specific oncolytic adenovirus and cisplatin on lung cancer cells. Oncol Lett.

[18] Chen S, Li YQ, Yin XZ, Li SZ, Zhu YL, Fan YY, Li WJ, Cui YL, Zhao J, Li X (2019). Recombinant adenoviruses expressing apoptin suppress the growth of MCF7 breast cancer cells and affect cell autophagy. Oncol Rep.

[19] Cui CX, Li YQ, Sun YJ, Zhu YL, Fang JB, Bai B, Li WJ, Li SZ, Ma YZ, Li X (2019). Antitumor effect of a dual cancer-specific oncolytic adenovirus on prostate cancer PC-3 cells. Urol Oncol.

[20] Zhang C, Zhou D (2016). Adenoviral vector-based strategies against infectious disease and cancer. Hum Vaccin Immunother.

[21] Danen-van Oorschot AA, van Der Eb AJ, Noteborn MH (2000). The chicken anemia virus-derived protein apoptin requires activation of caspases for induction of apoptosis in human tumor cells. J Virol.

[22] Maddika S, Booy EP, Johar D, Gibson SB, Ghavami S, Los M (2005). Cancer-specific toxicity of apoptin is independent of death receptors but involves the loss of mitochondrial membrane potential and the release of mitochondrial cell-death mediators by a Nur77-dependent pathway. J Cell Sci.

[23] Chaabane W, Appell ML (2016). Interconnections between apoptotic and autophagic pathways during thiopurine-induced toxicity in cancer cells: the role of reactive oxygen species. Oncotarget.

[24] Adams JM, Cory S (2007). The Bcl-2 apoptotic switch in cancer development and therapy. Oncogene.

[25] Bovellan M, Fritzsche M, Stevens C, Charras G (2010). Death-associated protein kinase (DAPK) and signal transduction: blebbing in programmed cell death. FEBS J.

[26] Espert L, Denizot M, Grimaldi M, Robert-Hebmann V, Gay B, Varbanov M, Codogno P, Biard-Piechaczyk M (2006). Autophagy is involved in T cell death after binding of HIV-1 envelope proteins to CXCR4. J Clin Invest.

[27] Yu L, Alva A, Su H, Dutt P, Freundt E, Welsh S, Baehrecke EH, Lenardo MJ (2004). Regulation of an ATG7-beclin 1 program of autophagic cell death by caspase-8. Science.

[28] Yu L, Lenardo MJ, Baehrecke EH (2004). Autophagy and caspases: a new cell death program. Cell Cycle.

[29] Kraft C, Peter M, Hofmann K (2010). Selective autophagy: ubiquitin-mediated recognition and beyond. Nat Cell Biol.

[30] Levine B, Klionsky DJ (2004). Development by self-digestion: molecular mechanisms and biological functions of autophagy. Dev Cell.

[31] Kirkin V, McEwan DG, Novak I, Dikic I (2009). A role for ubiquitin in selective autophagy. Mol Cell.

