# Supplementary material for: Apoptin mediates mitophagy and endogenous apoptosis by regulating the level of ROS in hepatocellular carcinoma
Source: Cell Commun Signal. 2022 Sep 1;20:134. doi: 10.1186/s12964-022-00940-1 (PMC9438158; doi:10.1186/s12964-022-00940-1)
Supplement: Supplementary file 2 — Additional file 1. Flow Graph of Changes in Apoptosis Level of Hepatoma Cells Treated with Apoptin. [file 12964_2022_940_MOESM2_ESM.docx]

**Supplementary information**

**Apoptin mediates mitophagy and endogenous apoptosis by regulating the level of ROS in hepatocellular carcinoma**

**Supplementary Figures and Legends**


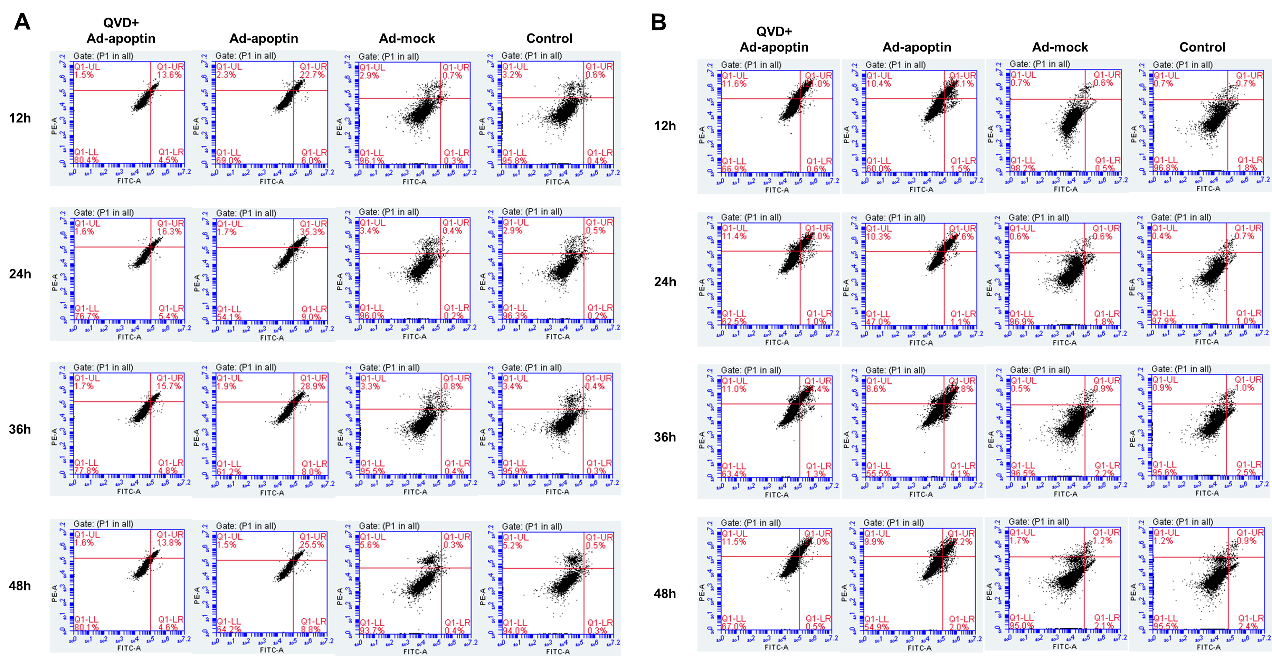


**Figure S1. Detection of Apoptosis Level of Hepatoma Cells.**

The apoptosis level of HepG-2 (A) and Huh-7 (B) cells were detected by Annexin V-FITC/PI staining. After the addition of the caspase inhibitor QVD (20 μM) to the cells infected with Ad-apoptin, the apoptosis level of HepG-2 and Huh-7 cells were significantly reduced.
